# Principles to guide research and policy on psychological well-being in remote island developing states in the South Pacific

**DOI:** 10.3389/fpsyt.2024.1325292

**Published:** 2024-03-20

**Authors:** Levente L. Orbán

**Affiliations:** School of Law and Social Sciences, The University of the South Pacific, Suva, Fiji

**Keywords:** South Pacific, mental health, resilience, human evolution, cross-cultural psychology

## Abstract

Adverse climatic changes around the globe and predictions of catastrophic and irreversible alteration in global weather patterns, temperature rise, and coast-line habitability require a careful examination of consequences on the resilience and mental health of people who will endure these changes. This paper is concerned with the South Pacific region. This geography has benefited from a relatively stable climate that is seen in the lush and vibrant natural world with many unique species of plants and animals exclusively found here. This paper examines the psychological profile of the people in the South Pacific using an evolutionary framework, and considers their local climate risks and lifestyle patterns with the aim of exploring possible mental health trajectories.

## 1 Introduction

Uncertainties due to deteriorating global climate, emerging novel diseases, and unpredictable geopolitical developments project negatively on people’s psychological well-being around the world (1–3). The psychological well-being profile of the South Pacific, composed of 12 Small Island Developing States (SIDS) with an overall population of 2.5 million people, is surprisingly similar to developed nations in some respects, and entirely divergent in others (4). Specifically, the consequences of poor mental health are identical in South Pacific SIDS and the developed world (1, 2, 4). For example chronic anxiety lead to lost productivity, poor quality of life, noncommunicable diseases, and psychiatric conditions in South Pacific SIDS and elsewhere (5). However, the way in which people in South Pacific SIDS respond to environmental threats is distinct from populations of industrialised countries: an accepting orientation towards climate uncertainties and a community driven response to adverse climatic events has protective effects on mental health. This conceptual paper will consider the distinct cultural, religious, and psychological profile of the South Pacific, the economic and technological landscape that presents unique regional challenges, and offers suggestions that could build on the region’s strengths.

## 2 Humans are climate adversity specialists

The environment in which humans evolved over the last 2.33 my (my: millions of years; mya: millions of years ago; kya: thousands of years ago) substantially differ from the environment that our species shaped for itself over the last 150 years (6). Infectious diseases, adverse climatic events, famine, and conflict are environmental pressures that are familiar to humans: they are selective forces that gave rise to modern humans. The modern world is safer today in relation to danger from predators, weather or infectious diseases. Record world population and crowding (7), our long lifespan and accompanying noncommunicable diseases (8), and legal and economic globalisation (9), have altered our living conditions in ways unknown to us before. These modern conditions have created a new set of selective pressures such as caloric abundance leading to obesity (10), diabetes and other diseases, information overload leading to thinking fallacies and behavioural biases (11), and high density living conditions leading to anxiety and depression among other conditions (12).

**Figure 1:**
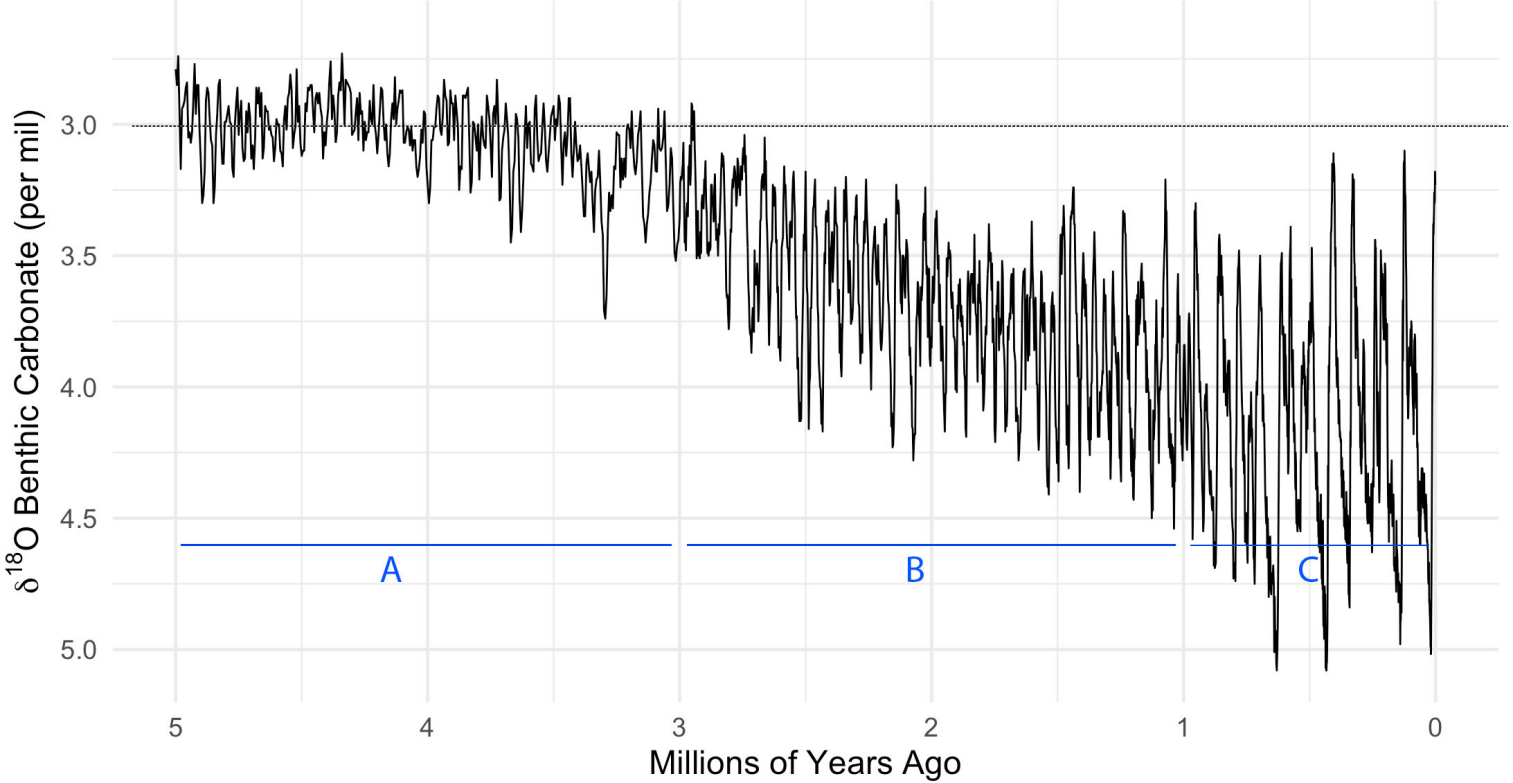
Reconstruction of the past 5 million years of climate history, based on oxygen isotope fractionation (serving as a proxy for the total global mass of glacial ice sheets. Glacial models using ice cores enables understanding Earth’s past climates. Cores from glaciers are used to analyse layers of ice that have accumulated over millennia. Oxygen isotope analysis helps infer past temperatures, with the ratio of oxygen isotopes varying with temperature. The physical properties of ice and trapped air bubbles within it provide insights into historical atmospheric conditions. The graph here indicates that temperature fluctuations have become increasingly amplified in the last 3 million years: **(A)** Temperature variation between 5.3 and 3 *mya* is **1x**. **(B)** Temperature variation between 3 and 1 *mya* is **2x** relative to **(A)**. **(C)** Temperature variation in the last 1 my is **2.8x** relative to **(A)**. This “LR04” stack is based on 38,000 individual *δ* ^18^ *O* measurements from 57 globally distributed sites. *δ* ^18^ *O* is a measure of the ratio of stable isotopes oxygen-18 and oxygen-16. The graph was constructed based on data published in Lisiecki et al. (14).

Climate reconstruction using isotope fractionation from glacial ice sheets reveals that the last 4 my have been characterised by an increasing level of environmental uncertainty (13, 14) (see **Figure 1**). Humans lived through ice ages several times, each period lasting thousands of years. As a proxy for cold periods, ice sheets advanced and receded from the north pole every 41 ky between 2.4 mya to 0.9 mya, and became more amplified and longer lasting since that time (15). Droughts lasting years and even decades were also familiar to humans in the last 100 ky (16, 17). Humans came to near extinction at least twice, once 900 kya with ∼1300 remaining individuals (18), and once 70 kya with ∼40 remaining breeding pairs (19, 20). Human intelligence and imagination in our species (*Homo sapiens*) likely emerged due to the necessity to solve complex adaptive problems in adverse climatic conditions (21).

Humans are facing severe climatic pressures again (22–24) as we approach a possible sixth mass extinction event (25, 26). The human population is the largest it has ever been (7), and under pressure from several environmental pressures simultaneously. The number of threats emerging in parallel, the compressed temporal scale coupled with the intensity at which each of these stressors is emerging, and the persistence at which these pressures are currently occurring may be unprecedented.

The climate of the South Pacific region diverges from average global patterns. While droughts and cyclones have been recorded in the past (27), the South Pacific’s equatorial location and surrounding large body of warm water has sheltered this region from the types of extreme temperature fluctuations and anomalous weather patterns detected elsewhere on the planet (28). At the same time, the future is more uncertain: sea level rise, beach erosion and increased temperature fluctuations could mean having to face a level of environmental stress that this region has not seen in the last 100 ky. This climate forecast in combination with the ripple effects of climate change elsewhere on the planet felt through economic shocks to this region together make a good reason to examine the impacts this climate adversity will have on the coping abilities of South Pacific SIDS populations.

## 3 From subsistence-based living to cash economy

Industrialised and economically diversified countries rely on specialised skill sets and advanced technologies to shelter from climate-related pressures. For example, advanced Heating, Ventilation and Air conditioning (HVAC) systems keep temperature constant in severely cold and hot weather (29). Climate change induced biological threats that endanger wildlife, livestock, agriculture, forestry, and public health can be mitigated through scientific research in health, agricultural and applied fields (e.g., vaccine development and dissemination) (30). Advanced and instant communication systems afforded by fibre optic, copper, wireless and even non-terrestrial Internet infrastructure facilitate access to information, and coordination of large groups of geographically dispersed people (31).

The South Pacific is less economically developed relative to industrialised countries, making it more exposed to climatic adversity and accompanying biological threats, and lack of access to the latest information to improve strategic decision making (32, 33). The current infrastructure in many South Pacific SIDS is riddled with power blackouts (34), Internet outages (35), water pressure drops and unsanitary water (36). Missing any one of these basic needs in a developed country could lead to significant shutdowns (setting aside system redundancies), drops in productivity, work absenteeism and adverse health outcomes, but this may not be the case in the South Pacific.

Psychological resilience refers to the ability to continue healthy functioning in the face of adverse events (37). In the face of cyclones, beach erosion or floods, the ability of people to come together, cope and rebuild are examples of psychological resilience. Accompanying this mental orientation is the concept of technological fault-tolerance: the ability for a system to continue functioning under suboptimal circumstances. For example, water storage tanks help cope with water outage; solar assisted hot water tanks provide low cost water heating even when power is out. Diesel generators and solar chargers provide electricity to the entire home, and subscribing to multiple Internet providers compensates for Internet outages due to faults in any one network. The psychological resilience of individuals in the South Pacific, and the widespread use of fault-tolerant technologies makes withstanding the variable performance of basic infrastructure tolerable. This fault-tolerant orientation towards technology also diverges from the way technology is utilised in economically diversified countries that consume technologies that increase centralisation and reliance on service providers: cloud services, subscriptions, technology leases and personal loans, all increase interdependence and can lead to cascading system failures (38).

Pacific SIDS have another significant strength: sustenance-based living is still in the recent past of the cultures. Whereas many cultures have experienced 10,500 years of agriculture, and over a hundred years of industrialisation, Pacific SIDS have experienced sustenance-based living as recently as 17th century (4, 39). The coronavirus (COVID-19) pandemic offered a rare insight into the way people treated lock-down, isolation and the consequent collapse of the tourism industry. In the absence of tourism income, communities whose primary occupation was at resorts reverted back to sustenance based living: fishing, gathering fruits and growing vegetables. Many people have not seen a salary for nearly a year while at the peak of the pandemic, and it did not lead to homelessness or significant economic adversity. On the contrary, the transition was straightforward and everyday life was less stressful, and many villages were spared from encountering the virus.

## 4 Cross-cultural and psychological factors

Cross-cultural psychology provides a useful organising principle for understanding mental health patterns and help distinguish the needs of the South Pacific relative to other cultures (40). According to cross-cultural dimensions defined by Hofstaede et al. (41, 42), the South Pacific can be characterised as having an interdependent identity construal, high tolerance for uncertainty and short-term orientation. An interdependent self-construal facilitates cooperation and produces a sense of emotional closeness, and better accuracy of predicting their own behaviour (less prone to overconfidence) (43–45). This trait is critical in times of adversity: a community pulling together to rebuild after the ravage of a cyclone is facilitated even in the absence of any support from government authorities or non-governmental organisations.

An anecdote shared by a former Fijian government official comes to mind. After a particularly damaging hurricane in 2016, many villages were left in ruins. A government convoy went to visit one of these villages to examine types of assistance to provide. As in many other villages, all houses collapsed with the exception of the only concrete structure in the village, a church. Officials feared what they would find in the aftermath of this hurricane, but when they arrived, they found the people gathered in the church where they played the guitar and sang together. To their even greater surprise, the entire convoy was offered lunch, prepared by the disaster struck villagers. In countless similar stories, villagers proceed to rebuild after disasters with minimal outside assistance.

Conversely, this same identity trait that facilitate cooperation and harmonious coexistence is associated with sharper in-group out-group boundaries (46). Harmony and cooperation comes at a cost to those seen in the out-group: they are kept at a distance and viewed as a potential adversary. This makes cooperation on a larger scale more challenging. Out-group boundaries naturally form along geographical, ethnic or religious lines, but they may form along political and ideological dimensions as well.

The maintenance of harmony and feelings of belonging are a great source of comfort for individuals with interdependent self-construal, but transgression from norms causes feelings of stress and shame (41) more so than those with individualistic self-construals. Consequently, speaking one’s mind, expressing personal opinions, and transgressions from the norm are avoided. Individual aspirations or achievements can be seen at the cost of group well-being.

High tolerance for uncertainty leads to accepting inherent uncertainties of life such as disease and death or changing economic fortunes, and even calamities due to climate change (41, 47, 48). This tolerance leads to better subjective well-being, and less rumination and stress over elements of life not within one’s control. However, combined with a short-term orientation means focus is on past-events and the present time, which leads to personal steadiness, emphasis on traditions and valuing service to others. However, making commitments to long-term strategic plans and persistently building towards those plans takes less priority.

Some nations in the region such as Fiji are multi-ethnic (3 ethnic groups comprise 90% of the population) whereas most others, such as Tonga, Kiribati or Samoa are homogeneous (over 90% of people a single ethnicity) (4). All nations in the South Pacific are religious and have largely embraced Christianity, but groups that arrived with colonisation (e.g., labour, merchants) retained their religion of origin (Hinduism, Islam are prominent in Fiji). Limited immigration to these regions facilitate social networks in which relationship longevity is high and stable, measured in decades or lifetimes, and social network density is optimal: family and friends are within functional proximity (49). Crowding such as in large cities can be an isolating experience in urban centres (50). The largest city in the South Pacific is Suva, with a population of merely 93,970 (2017 estimate). Despite relatively low urbanisation at the present time, this is an amplifying trend and a direct cause of distress for individuals.

Fiji is unique in the region in its multicultural character. The conception of multiculturalism is different from countries with permissive immigration policies such as the United States, Germany, the United Kingdom or Canada. Most immigration into Fiji occurred in the 18th and 19th centuries (4, 51). Presently, immigration is limited to work permit holders and returning expatriates (52). A consequence of such limited multiculturalism is learning and convergence between cultures that arrived before (53). The Fijian native i-Taukei and Rotuman cultures have had a century of conflict and harmony with Indians who comprised much of the immigration in the 19th century (54). In terms of religion, most island nations show over 95% engagement with Christianity, except for Fiji where Christianity comprises 65% of the population (Hinduism (20%) and Islam (6%)). This is unlike many western developed nations where Christianity is on the decline, ranging between 50-70% in countries with a traditionally Christian character.

The impact of religion, Christianity in particular, on mental health is a subject of considerable interest. Some individuals find solace, purpose, and social support within religious communities, which can have positive effects on their mental health. For instance, participation in religious activities may offer a sense of belonging and emotional comfort, reducing symptoms of anxiety and depression (55). Moreover, the practice of prayer or meditation associated with Christianity may promote relaxation and stress reduction, contributing to improved mental resilience (56).

## 5 Conclusions

This psychological, cultural and religious context creates mental health vulnerabilities in areas that are different from industrialised nations. Stress due to consequences of changing lifestyle such as leaving one’s village and not finding a sense of belonging in cities, changing of traditional social hierarchies such as the weakened role of senior men, expanding roles of women, and the multitude of social transgressions employers demand of workers are amplified in this region (57, 58). My suspicion is that anxiety and depression is largely due to repeated social defeat associated with this shift, and isolation and loneliness while adopting an independent identity in cities (59).

Diagnostics, monitoring and treatment of psychiatric disorders is a significant challenge in the South Pacific (60). Low economic diversification, the absence of governing bodies for regulating mental health treatment, and remote, inaccessible geographies with poor infrastructure present difficult problems (61). The number of clinicians is low and specialised postgraduate training is scarce even in urban areas. In terms of infrastructure for telehealth, cell tower coverage is weak (outside of the Fiji coast), copper lines (broadband, telephony) do not penetrate villages, and power supply is intermittent, making remote service delivery and telehealth impossible. Focusing on leapfrogging opportunities such as non-terrestrial internet networks (e.g., StarLink (SpaceX, Austin, United States of America) low orbit satellites), solar charged and computing could fast-track the deployment of international expertise even in the most remote locations.

Mental health awareness in villages is not conceptualised in ways familiar in individualistic cultures. Anxiety, depression, trauma are addressed through strong social networks and helpful coping strategies (61, 62). Frequent contact with family and close friends, sharing in each other’s grief and happiness through singing, talking, and working together protect against many of the ailments common in individualistic cultures. These social behaviours are often ritualised through traditional healing modalities such as the use of herbs, yaqona, massage. Mental health services are not existent for these conditions, but are not a priority. Psychoeducation to recognise hallmarks of psychiatric conditions such as psychosis, schizophrenia or bipolar disorders would be useful (63).

People of South Pacific SIDS show superior resilience towards direct climate related impacts, but significant vulnerability towards transition into urbanised lifestyle and economic development, and the ripple effects of distant climate change calamities to the economic system (e.g., inflation, workload, financial hardship). Mental health services could focus on aiding the transition towards an urbanised and economy based lifestyle: loss of social networks, identity confusion, and repeated social defeat. Older males are of particular concern as they stand to lose the most in this transition. Due to a fall in social status, lacking relevant skills in an urban environment and significant time and financial costs of retraining, they are at an increased risk of turning towards domestic violence or committing suicide.

## Author contributions

LO: Conceptualization, Formal Analysis, Writing – original draft, Writing – review & editing.

## References

[1] Claveria O. Global economic uncertainty and suicide: Worldwide evidence. Soc Sci Med. (2022) 305:115041. doi: 10.1016/j.socscimed.2022.115041 35598442

[2] Mezzina R Gopikumar V Jenkins J Saraceno B Sashidharan SP. Social vulnerability and mental health inequalities in the “syndemic”’: Call for action. Front Psychiatry. (2022), 13. doi: 10.3389/fpsyt.2022.894370 PMC9210067 35747101

[3] Sampogna G Pompili M Fiorillo A. Mental health in the time of covid-19 pandemic: A worldwide perspective. Int J Environ Res Public Health. (2022) 19:1–5. doi: 10.3390/ijerph19010161 PMC8750501 35010419

[4] Rich GJ Ramkumar NA. International and cultural psychology: psychology in oceania and the caribbean. Washington, USA: Springer (2022). doi: 10.1007/978-3-030-87763-7

[5] De Beurs E Beekman ATF van Balkom AJLM Deeg DJH van Dyck R van Tilburg W. Consequences of anxiety in older persons: its effect on disability, well-being and use of health services. psychol Med. (1999) 29:583–93. doi: 10.1017/s0033291799008351 10405079

[6] Antón SC. Early homo: Who, when, and where. Curr Anthropology. (2012) 53:S278–98. doi: 10.1086/667695

[7] Cohen J. Human population: The next half century. Science. (2003) 302:1172–5. doi: 10.1126/science.1088665 14615528

[8] Wickramasinghe K Mathers JC Wopereis S Marsman DS Griffiths JC. From lifespan to healthspan: the role of nutrition in healthy ageing. J Nutr Sci. (2020) 9:1–10. doi: 10.1017/jns.2020.26 PMC7550962 33101660

[9] Vasyliev S Epryntsev P Rekunenko T. Legal globalisation and economic systems. Baltic J Economic Stud. (2023) 9(4):50–7. doi: 10.30525/2256-0742/2023-9-4-50-57

[10] Bellisari A. Evolutionary origins of obesity. Obes Rev (2008) 9:165–80. doi: 10.1111/j.1467-789X.2007.00392.x 18257754

[11] Keller KL Staelin R. Assessing biases in measuring decision effectiveness and information overload. J Cons Res (1989) 15(4):504–8.

[12] Cermakova P Csajbók Z. Household crowding in childhood and trajectories of depressive symptoms in mid-life and older age. J Affect Dis (2023) . 340:456–61. doi: 10.1016/j.jad.2023.08.056 37573892

[13] Zachos J Pagani M Sloan E Thomas L Billups K. Trends, rhythms, and aberrations in global climate 65 ma to present. Science. (2001) 292:686–93. doi: 10.1126/science.1059412 11326091

[14] Lisiecki LE Raymo ME. A pliocene-pleistocene stack of 57 globally distributed benthic *δ* ^18^ *o* records. Paleoceanography. (2005) 20:PA1003. doi: 10.1029/2004PA001071

[15] Hewitt G. The genetic legacy of the quaternary ice ages. Nature. (2000) 405:907–13. doi: 10.1038/35016000 10879524

[16] Meko D Stockton CW Boggess WR. The tree-ring record of severe sustained drought. Water Resour Bull. (1995) 31:789–801. doi: 10.1111/j.1752-1688.1995.tb03401.x

[17] Peñuelas J Lloret F Montoya R. Severe drought effects on mediterranean woody flora in Spain. For Sci. (2001) 47:214–8.

[18] Hu W Hao Z Du P Vincenzo FD Manzi G Cui J. Genomic inference of a severe human bottleneck during the early to middle pleistocene transition. Science. (2023) 381(6661):979–84. doi: 10.1126/science.abq7487 37651513

[19] Wilson EO. The social conquest of earth. New York, USA: W. W. Norton Co (2012).

[20] Osipov S Stenchikov G Tsigaridis K LeGrande AN Bauer SE Fnais M. The toba supervolcano eruption caused severe tropical stratospheric ozone depletion. Commun Earth Environ. (2021) 2:1–7. doi: 10.1038/s43247-021-00141-7

[21] Templer DI Stephens. JS. The relationship between iq and climatic variables in african and eurasian countries. Intelligence. (2014) 46:169–78. doi: 10.1016/j.intell.2014.06.001. D. I.

[22] Hammond WM Williams AP Abatzoglou JT Adams HD Klein T López R. Global field observations of tree die-off reveal hotter-drought fingerprint for earth’s forests. Nat Commun. (2022) 13:1–11. doi: 10.1038/s41467-022-29289-2 PMC8983702 35383157

[23] Pörtner H-O Roberts D Tignor M Poloczanska E Mintenbeck K Alegría A. *IPCC 2022*: climate change 2022: impacts, adaptation, and vulnerability. In: Contribution of working group II to the sixth assessment report of the intergovernmental panel on climate change. Cambridge, UK: Cambridge University Press (2022). doi: 10.1017/9781009325844

[24] Müller J Hothorn T Yuan Y Seibold S Mitesser O Rothacher J. Weather explains the decline and rise of insect biomass over 34 years. Nature. (2023). doi: 10.1038/s41586-023-06402-z 37758943

[25] Barnosky AD Matzke N Tomiya S Wogan GOU Swartz B Quental TB. Has the earth’s sixth mass extinction already arrived? Nature. (2011) 471:51–7. doi: 10.1038/nature09678 21368823

[26] Naggs F. Saving living diversity in the face of the unstoppable 6th mass extinction: a call for urgent international action. J Population Sustainability. (2017) 1(2):67–81. doi: 10.3197/jps.2017.1.2.67

[27] Higgins PA Palmer JG Andersen MS Turney CSM Johnson F. Extreme events in the multi-proxy south pacific drought atlas. Climate Change. (2023) 176:105. doi: 10.1007/s10584-023-03585-2

[28] Herrando-Moraira S Nualart N Galbany-Casals M Garcia-Jacas N Ohashi H Matsui T. Climate stability index maps, a global high resolution cartography of climate stability from pliocene to 2100. Sci Data. (2022) 9(48):1–12. doi: 10.1038/s41597-022-01144-5 PMC8831633 35145118

[29] Argyroudisa SA Mitoulis SA Chatzi E Baker JW Brilakis I Gkoumas K. Digital technologies can enhance climate resilience of critical infrastructure. Climate Risk Manage. (2022) 35:100387. doi: 10.1016/j.crm.2021.100387

[30] More AF Loveluck CP Clifford H Korotkikh EV Kurbatov AV McCormick M. The impact of a six-year climate anomaly on the “spanish flu” pandemic and wwi. GeoHealth. (2020) 4:1–8. doi: 10.1029/2020GH000277 PMC7513628 33005839

[31] Philip G Hodge R. Disaster area architecture: telecommunications support to disaster response and recovery. In: Proceedings of MILCOM ‘95. San Diego, USA (1995). doi: 10.1109/milcom.1995.483644

[32] International Monetary Fund. International monetary fund: world economic outlook database. Washington, USA: Tech. rep (2019).

[33] United Nations. GDP/breakdown at current prices in National currency (all countries). United Nations: Tech. rep. (2023).

[34] Mamun KA Islam FR. (2016). Reliability evaluation of power network: A case study of Fiji islands, in: Australasian Universities Power Engineering Conference, AUPEC, . doi: 10.1109/AUPEC.2016.7749359

[35] Whelan R. Use of ict in education in the south pacific: findings of the pacific elearning observatory. Distance Educ. (2008) 29(1):53–70. doi: 10.1080/01587910802004845

[36] Weber E. Water in the pacific islands: Case studies from Fiji and Kiribati. In: Gover VI, editor. Water: A source of conflict or cooperation. Hamilton, Canada: Science Publishers (2007).

[37] Southwick SM Bonanno GA Masten AS Panter-Brick C Yehuda R. Resilience definitions, theory, and challenges: interdisciplinary perspectives. Eur J Psychotraumatol. (2014) 5:1–14. doi: 10.3402/ejpt.v5.25338 PMC4185134 25317257

[38] Little RG. Controlling cascading failure: Understanding the vulnerabilities of interconnected infrastructures. J Urban Technol. (2002) 9(1):109–23. doi: 10.1080/106307302317379855

[39] Balkrishna A Sharma G Sharma N Kumar P Mittal R Parveen R. Global perspective of agricultural systems: From ancient times to the modern era. In: Balkrishna A, editor. Sustainable agriculture for food security: A global perspective. Palm Bay, USA: CRC Press (2022).

[40] Bass JK Bolton P Murray LK. Do not forget culture when studying mental health. Lancet. (2007) 370:918–9. doi: 10.1016/s0140-6736(07)61426-3 17869621

[41] Hofstaede G. Dimensionalizing cultures: The hofstaede model in context. Online Readings Psychol Culture. (2011) 2(1):1–26. doi: 10.9707/2307-0919.1014

[42] Hofstaede GH. *Culture’s consequences: International differences in work-related values* (Sage). London, UK (1980).

[43] Li HZ Zhang Z Bhatt G Yum Y-O. Rethinking culture and self-construal: China as a middle land. J Soc Psychol. (2006) 146(5):591–610. doi: 10.3200/SOCP.146.5.591-610 17042404

[44] Balcetis E Dunning D Miller RL. Do collectivists know themselves better than individualists? cross-cultural studies of the holier than thou phenomenon. J Pers Soc Psychol. (2008) 95(6):1252–67. doi: 10.1037/a0013195 19025282

[45] Tu K Chen S Mesler RM. Trait self-construal, inclusion of others in the self and self-control predict stay-at-home adherence during covid-19. Pers Individ Dif. (2021) 175:110687. doi: 10.1016/j.paid.2021.110687 34848903 PMC8613711

[46] Fischer R Derham C. Is in-group bias culture-dependent? a meta-analysis across 18 societies. SpringerPlus. (2016) 5:1–9. doi: 10.1186/s40064-015-1663-6 PMC4723375 26839763

[47] Neher F Miola A. Culture and resilience. JRC Tech Rep. (2016).

[48] Lenton TM Boulton CA Scheffer M. Resilience of countries to covid-19 correlated with trust. Sci Rep. (2022) 12(75):1–15. doi: 10.1038/s41598-021-03358-w PMC8738739 34992222

[49] Kern ML Porta SSD Friedman HS. Lifelong pathways to longevity: Personality, relationships, flourishing, and health. J Pers. (2013) 82(6):472–84. doi: 10.1111/jopy.12062 23927423

[50] Jedwab R Loungani P Yezer A. Comparing cities in developed and developing countries: Population, land area, building height and crowding. Regional Science Urban Economics. (2021) 86:103609. doi: 10.1016/j.regsciurbeco.2020.103609

[51] Prasad KK. The gujaratis of Fiji 1900-1945: A study of an Indian immigrant trader community. Ph.D. thesis. Vancouver, Canada: University of British Columbia (1978) 46(133):133–59.

[52] International Organization for Migration. Migration in the republic of Fiji: A country profile 2020. Geneva, Switzerland: International Organization for Migration. Tech. rep. (2020).

[53] Minkov M Hofstaede G. Is national culture a meaningful concept? : Cultural values delineate homogeneous national clusters of in-country regions. Cross-Cultural Res. (2011). doi: 10.1177/1069397111427262

[54] Chand S. The political economy of Fiji: Past, present, and prospects. Round Table. (2015) 104(2):199–208. doi: 10.1080/00358533.2015.1017252

[55] Smith T McCullough. ME Poll J. Religiousness and depression: Evidence for a main effect and the moderating influence of stressful life events. psychol Bull. (2003) 116:614–36. doi: 10.1037/0033-2909.129.4.614 12848223

[56] Koenig HG. Religion, spirituality, and health: The research and clinical implications. International Scholarly Research Network Psychiatry (2012), 1–33. doi: 10.5402/2012/278730 PMC3671693 23762764

[57] Gely R Bierman L. Love, sex and politics? sure. salary? no way: Workplace social norms and the law. Berkely J Employment Labor Law. (2004) 25:164.

[58] Santos MS. Affective adaptation of social norms in workplace design. Ph.D. thesis. London, UK: Imperial College London (2013).

[59] Jones P Lea JP. What has happened to urban reform in the island pacific? some lessons from Kiribati and Samoa. Pacific Affairs. (2007) 80:473–91. doi: 10.5509/2007803473

[60] Puamau ES. Utilisation review of first admissions in 2002: St giles psychiatric hospital, suva, Fiji. Pacific Public Health. (2006) 13(3):79–88. 18181394

[61] Roberts G Leckie J Chang O. The history of mental health in Fiji. In: Minas H Lewis M, editors. Mental health in asia and the pacific, international and cultural psychology. Heidelberg, Germany: Springer (2017).

[62] Alam M Ali MF. Health and wellbeing and quality of life in the changing urban environment: Challenges and government response in Fiji island. J Positive School Psychol. (2022) 6:2853–63.

[63] Donker T Griffiths KM Cuijpers P Christensen H. Psychoeducation for depression, anxiety and psychological distress: a meta-analysis. BMC Med. (2009) 7(79):1-9. doi: 10.1186/1741-7015-7-79 PMC2805686 20015347

